# *In situ* Metabolomics of Metabolic Reprogramming Involved in a Mouse Model of Type 2 Diabetic Kidney Disease

**DOI:** 10.3389/fphys.2021.779683

**Published:** 2021-11-30

**Authors:** Bai Linnan, Wang Yanzhe, Zhang Ling, Liu Yuyuan, Chen Sijia, Xie Xinmiao, Li Fengqin, Wang Xiaoxia

**Affiliations:** Department of Nephrology, Shanghai Tongren Hospital, Shanghai Jiao Tong University School of Medicine, Shanghai, China

**Keywords:** mass spectrometry imaging, metabolomics, diabetic kidney disease, metabolic pathways, metabolic reprogramming

## Abstract

The *in situ* metabolic profiling of the kidney is crucial to investigate the complex metabolic reprogramming underlying diabetic kidney disease (DKD) and to allow exploration of potential metabolic targets to improve kidney function. However, as the kidney is a highly heterogeneous organ, traditional metabolomic methods based on bulk analysis that produce an averaged measurement are inadequate. Herein, we employed an *in situ* metabolomics approach to discover alternations of DKD-associated metabolites and metabolic pathways. A series of histology-specific metabolic disturbances were discovered *in situ* using airflow-assisted desorption electrospray ionization mass spectrometry imaging (AFADESI–MSI). In combination with integrated metabolomics analysis, five dysfunctional metabolic pathways were identified and located in the kidneys of type-2 DKD mice simultaneously for the first time, including taurine metabolism, arginine and proline metabolism, histidine metabolism, biosynthesis of unsaturated fatty acids, and fatty acid degradation pathways. As crucial nodes of metabolic pathways, five dysregulated rate-limiting enzymes related to altered metabolic pathways were further identified. These findings reveal alternations from metabolites to enzymes at the molecular level in the progression of DKD and provide insights into DKD-associated metabolic reprogramming.

## Introduction

The prevalence of diabetic kidney disease (DKD) as a major complication of diabetes is increasing rapidly, with approximately 30% of cases of end-stage renal disease worldwide in 2015 being due to diabetes (Cheng et al., 2021). As a highly metabolic organ, the kidney is vulnerable to systemic metabolic disorders, such as chronic hyperglycemia, dyslipidemia, and obesity, which may cause renal metabolic disturbance and lead to renal dysfunction (Kalim and Rhee, 2017; Forbes and Thorburn, 2018). Although strict measures can be introduced, such as blood glucose and lipid control, renal function typically remains poor (known as the “metabolic memory” phenomenon), partially because of our incomplete understanding of the intricate metabolic alternations involved in the progression of DKD (Zoungas et al., 2017; Anders et al., 2018).

Metabolic reprogramming of small-molecular compounds is closely associated with DKD, which is itself a complex metabolic disease (Hasegawa and Inagi, 2021). Metabolomics technology, an effective systematic analysis of metabolites detected within biological samples, is particularly appropriate for DKD studies and has been used in cases, such as the identifying of novel biomarkers and the demonstration of pathogenesis (Kalim and Rhee, 2017). By using liquid chromatography–mass spectrometry (LC–MS)-, gas chromatography–mass spectrometry (GC–MS)-, and capillary electrophoresis-mass spectrometry (CE–MS)-based metabolomics, studies have discovered several metabolic reprogramming pathways related to DKD (Sas et al., 2016; Hasegawa et al., 2020; Sha et al., 2020). Although these studies have improved our understanding of the disruption of various metabolites involved in DKD, the methodology was based on bulk tissue techniques in which the metabolites were extracted from tissue homogenates, resulting in averaged measurements with no spatial localization information (Andersen et al., 2021). As a highly heterogeneous organ, the kidney is composed of the cortex, medulla, and central pelvis, and each of these structures contains specialized functional units to achieve different fundamental biological effects. Therefore, metabolic analysis based on a tissue-specific understanding of the kidney is crucial to comprehend the complex pathological processes underlying DKD.

Substantial efforts have been made in molecular image technology. Mass spectrometry imaging (MSI) has developed rapidly in recent years and can be used to identify multiple metabolites on a spatial level with high sensitivity and resolution, and without the need for labeling (Unsihuay et al., 2021; Zullig and Kofeler, 2021). For example, matrix-assisted laser desorption ionization mass spectrometry imaging (MALDI–MSI) has been used for the *in situ* analysis of metabolites in DKD research, although this method usually requires a high-vacuum environment and matrix assistance for ionization (Tanaka et al., 2018). Desorption electrospray ionization mass spectrometry imaging (DESI–MSI) is a powerful tool that has been used for lipid detection in the renal cortex of a type-1 diabetes mellitus (T1DM) mouse model, whereas reveals relatively low spatial resolution (Zhang et al., 2020a). Similar to DESI–MSI, tissue identified by airflow-assisted desorption electrospray ionization mass spectrometry imaging (AFADESI–MSI) exhibits great specificity, high sensitivity, and a wide coverage for spatial metabolomics since it can map a large number of metabolites located in diverse metabolic pathways (He et al., 2018; Sun et al., 2019; Wang et al., 2020b). However, the high-throughput discovery of type 2 DKD metabolic alterations has not been previously reported.

In the metabolic network, rate-limiting enzymes regulate the rate and direction of metabolic pathway processes, and the composition of metabolites in the tissue may reflect enzyme capacities (Ighodaro, 2018; Park et al., 2019). Growing evidence indicates that alternations in enzymes are related to the establishment of a “metabolic memory,” since various metabolic pathways are altered by the activation or inhibition of the expression of specific enzymes in the context of diabetes (Reddy et al., 2015; Qi et al., 2017; Anders et al., 2018; Gordin et al., 2019). This provides new insights for the development of potential biomarkers and drug targets for DKD.

**Figure 1:**
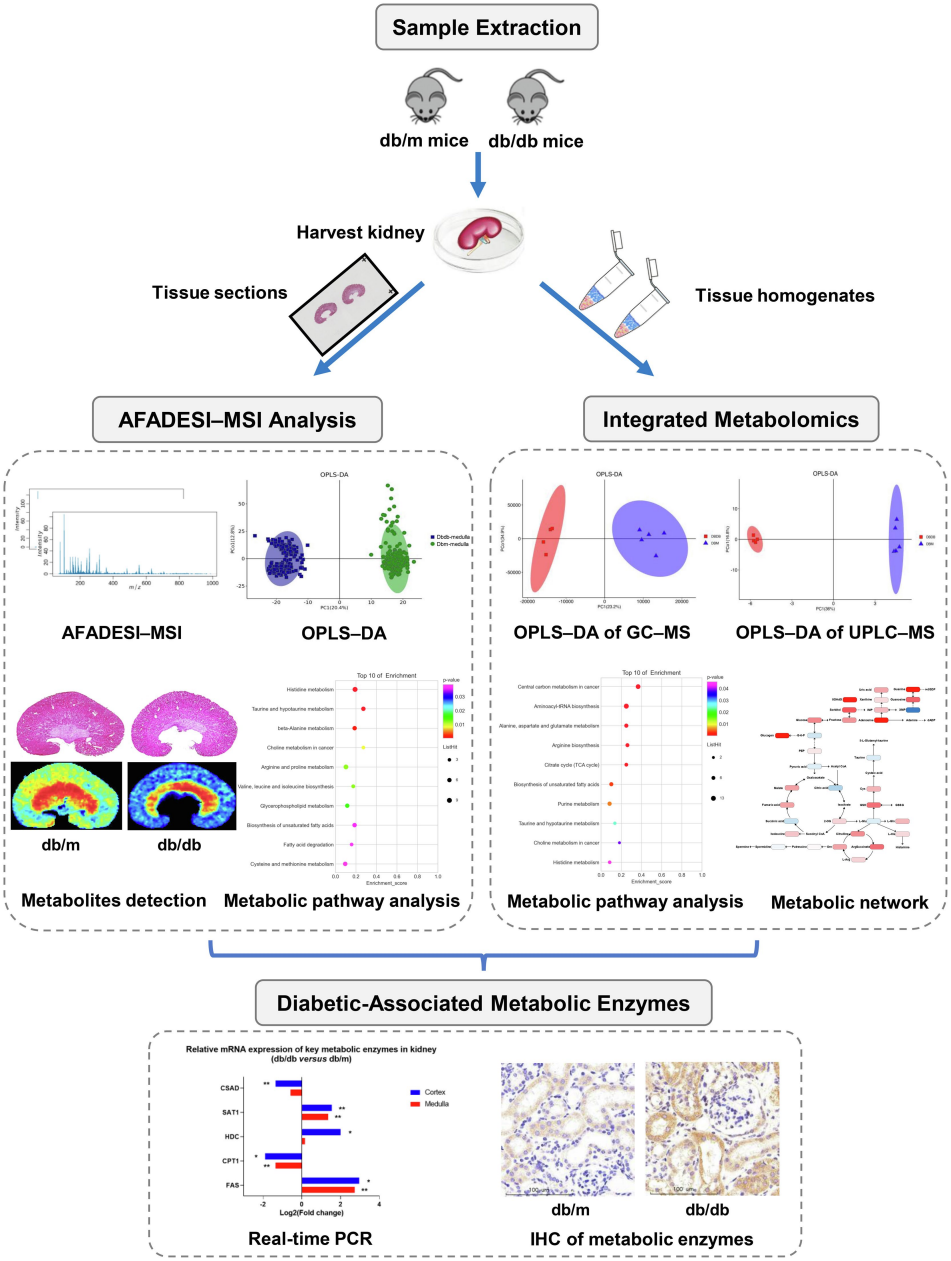
Research strategy employed in the present study for the *in situ* metabolomics of metabolic reprogramming involved in a mouse model of type 2 diabetic kidney disease.

Here, we describe a high-throughput *in situ* study of the metabolites and enzymes in metabolic reprogramming involved in DKD. A schematic workflow is summarized in Figure 1. First, a BKS-db/db mouse, a mature type-2 diabetes mellitus (T2DM) mouse model, was used and verified by biochemical and histopathological tests. Then, AFADESI–MSI was used for a histology-specific molecular characterization of the metabolites. Orthogonal projection of latent structure discriminant analysis (OPLS–DA) was performed to select discriminating metabolites between the diabetic group and control group, and then, metabolic pathway analysis was conducted to screen *in situ* metabolic pathway reprogramming, revealing potential diabetic-associated metabolic enzymes. We also examined the whole renal tissue by using integrated metabolomics of GC–MS and ultra-performance liquid chromatography–mass spectrometry (UPLC–MS) to acquire the total amount of altered metabolites, while mapped these metabolites located in metabolic pathways for comprehensive understanding of metabolic reprogramming. Finally, real-time polymerase chain reaction (PCR) and specific immunohistochemistry (IHC) staining were employed to validate the altered mRNA and spatial expression of the potential metabolic enzymes, respectively. Collectively using this approach, the spatial distribution and alternations of diabetic-associated metabolites and enzymes were visualized, and the reprogramming of metabolic pathways was identified, providing metabolism-based insights into the mechanism of the progression of DKD.

## Materials and Methods

### Animals and Grouping

Six-week-old male BKS-db/db and db/m mice were purchased from Cavens Laboratory Animals Co., Ltd. (Changzhou, China. License Number: SCXK 2016-0010). They were housed at room temperature (22±2°C) under a 12-h dark/12-h light cycle and fed a standard chow diet *ad libitum* until 24weeks of age. Body weight was measured monthly, and overnight fasting abdominal blood glucose was tested using a tail nick and glucometer (Roche Accu-Chek Advantage Meter). A 24-h urine samples were collected from non-fasting mice placed in metabolic cages at 12 and 24weeks of age, respectively. After fasting for 12h, the mice were euthanized at 24weeks of age with 1% pentobarbital, the blood samples were collected through the angular vein and centrifuged at 3500*g* for 10min at 4°C to acquire serum. Their kidneys were immediately extirpated and flash-frozen in liquid nitrogen for 5s, then transferred to cryogenic vials and kept at −80°C until further analysis. The left kidneys were used for AFADESI–MSI analysis (db/m, *n*=2; db/db, *n*=2) or integrated metabolomics analysis (db/m, *n*=5; db/db, *n*=4), the contralateral kidneys were used for histopathological examination and real-time PCR experiments (db/m, *n*=7; db/db, *n*=6).

The experimental procedures were approved by the Animal Care and Use Committee of Shanghai Jiao Tong University (Shanghai, China).

### Biochemical Analysis and Histopathological Examination

Analyses of serum were performed by the Servicebio Technology Co., Ltd. (Wuhan, China). The concentration of urine albumin was detected using a mouse albumin ELISA kit (Abcam), then multiplied by the 24-h urine volume of each mouse to calculate the 24-h urine albumin excretion.

For histopathological examination, the right kidneys were split into two parts in an ice bath. One part was fixed in 10% formalin and sectioned at a thickness of 3μm using a Leica CM1860 cryostat (Leica Microsystem Ltd., Wetzlar, Germany) at −20°C for periodic acid–Schiff (PAS) staining and IHC examination. Portions of the renal cortex (1×mm^3^ in volume) were dissected and maintained in 2.5% glutaraldehyde for analysis using a transmission electron microscope. For IHC staining, the tissue sections were incubated overnight at 4°C with specific primary antibodies (Supplementary Table S1); then, a PV-9000 two-step immunohistochemical kit (Zhongshan Goldenbridge Biotechnology Ltd. Co., Beijing, China) was used, followed by counterstaining with Mayer hematoxylin and dehydration. For the quantification of glomerular areas, we took a pathological picture from the PAS staining image of each sample and measured 20 glomerular areas, respectively, using a NanoZoomer Digital Pathology Image Viewer (NanoZoomer 2.0, Hamamatsu Photonics, Hamamatsu City, Japan).

### AFADESI–MSI Analysis of Tissue Sections

#### Sample Preparation

The left kidneys were fixed to the chuck in a Leica CM1860 cryostat at −20°C with optimal cutting temperature compound (OCT; Sakura Finetek, Torrance, CA, United States). A minimal amount of OCT was used, and the regions that were sectioned were not touched, in order to avoid the effect of OCT. The tissues were sectioned carefully at a thickness of 10μm and thaw-mounted into glass slides, then stored at −80°C until MSI and hematoxylin–eosin (H&E) staining. Before AFADESI–MSI analysis, the slides were dried in a vacuum desiccator for 30min. H&E staining of the adjacent tissue sections was performed to reveal histological differentiation.

#### AFADESI–MSI Analysis

AFADESI–MSI analysis was performed as previously reported (Sun et al., 2019). In brief, an AFADESI–MSI system with a lab-made AFADESI ion source and a Q-OT-qIT hybrid mass spectrometer (Orbitrap Fusion Lumos; Thermo Fisher Scientific, United States) was employed for tissue molecular profiling with a spatial resolution of 100μm. The MSI analysis was carried out in both the positive and negative ion mode from a mass-to-charge ratio (m/z) of 70 to 1,000, with high mass accuracy (<5ppm mass error) and a high mass resolution of 70,000. Nitrogen (0.6MPa) was used as spray gas, and a mixture of acetonitrile and water (8:2, v/v, 5μl/min) was used as spray solvent. The sprayer voltages were set at 7.0kV in the positive ion mode and at −7.0kV in the negative ion mode, and the capillary temperature was 350°C. The kidney tissue section was continuously scanned at a rate of 200μm/s in the *x*-direction, separated by a 100μm step in the *y*-direction.

#### Data Processing

The methodology used for AFADESI–MSI data analysis has previously been described (Wang et al., 2020b). Briefly, raw data sets were converted and loaded into high-performance imaging software (MassImager 2.0, Beijing, China) to rebuild the ion images. After manually delineating the regions of interest (ROIs) through matching H&E stained images of the adjacent tissue sections, the ion intensity of region-specific MS profiling was generated and imported into Markerview 1.2.1 software (AB SCIEX, Toronto, Ontario, Canada) for background deduction, peak picking, and peak alignment. Afterward, to acquire histology-specific discriminating metabolites, the relative intensities of ions were performed by using OPLS–DA.

### Integrated Metabolomics Analysis

As previously mentioned, the integrated metabolomics analysis in this study was conducted by GC–MS and UPLC–MS. The methodology has previously been described (Cao et al., 2020). In brief, GC–MS analysis was conducted using an Agilent 7890B gas chromatography system coupled to an Agilent 5977A MSD system (Agilent Technologies Inc., CA, United States). UPLC–MS analysis was performed using a Dionex Ultimate 3,000 RS UHPLC system fitted with Q-Exactive quadrupole-Orbitrap mass spectrometer equipped with a heated electron spray ionization (ESI) source (Thermo Fisher Scientific, Waltham, MA, United States) to analyze metabolic profiling in both positive and negative ion modes. The detailed sample preparation, workflow of GC–MS, and UPLC–MS analysis of kidney homogenates are described in the Supplemental methods.

### Total RNA Extraction and Real-Time PCR

The kidney samples were dissected carefully to separate the cortex and medulla on ice. Total RNA was extracted from the samples using TRIzol (Invitrogen, Carlsbad, CA), and the cDNA was synthesized using the reverse transcript reagents (Takara, Otsu, Japan). Real-time PCR was conducted using an SYBR Green RT-qPCR Kit (ABclonal, Wuhan, China) and a 7,500 Real-Time PCR System (Applied Biosystems, Waltham, MA). The relative gene expression levels were calculated using the 2ΔΔCT method and analytical data were adjusted with the mRNA expression of β-actin as an internal control. Specific primers used are listed in Supplementary Table S2.

## Results

### Background Data of db/db Mice

**Figure 2:**
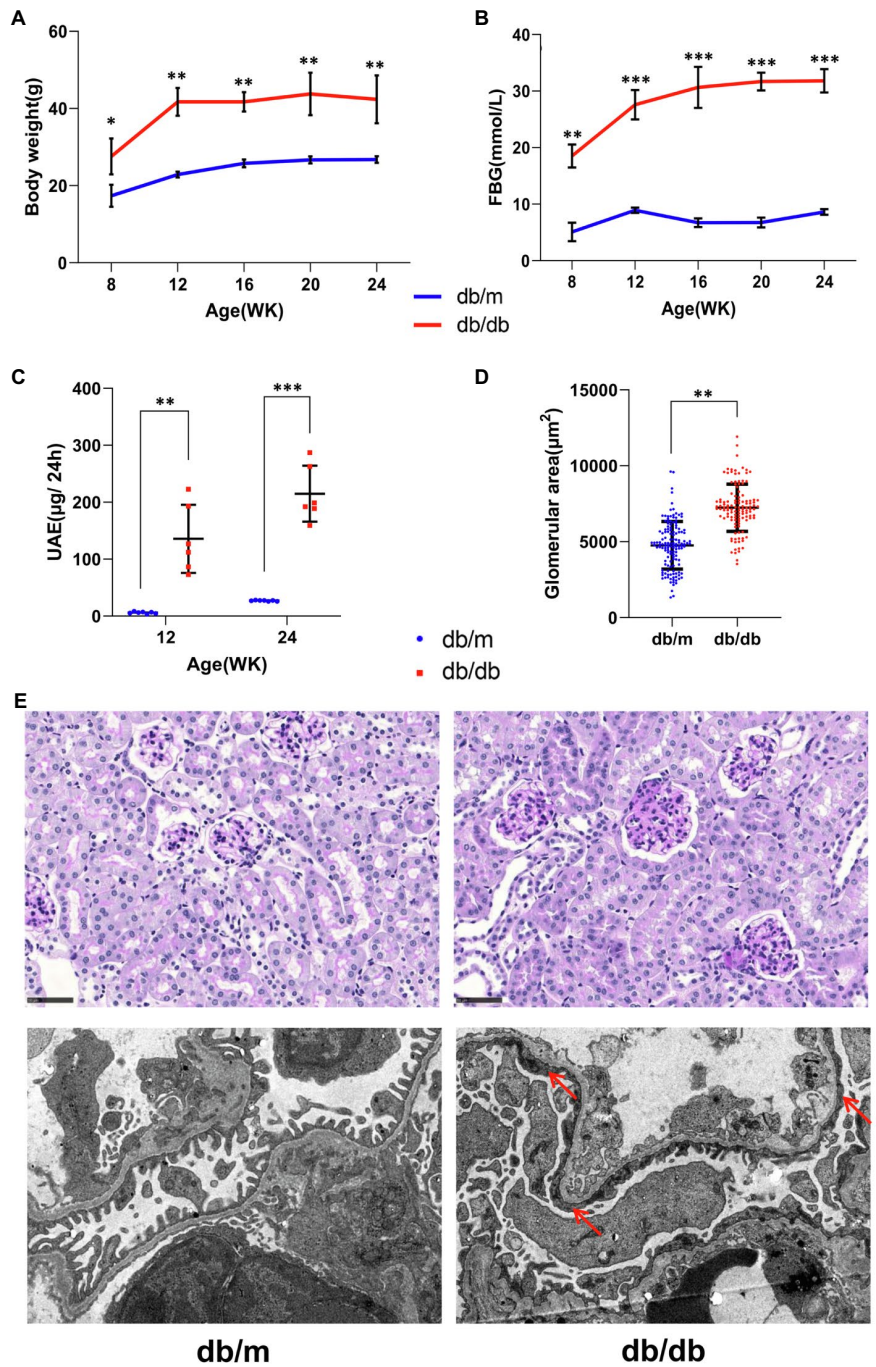
Basic biochemical parameters and pathologies from db/m (*n*=7) and db/db (*n*=6) mice. **(A)** Body weight, **(B)** overnight fasting abdominal blood glucose (FBG), and **(C)** 24-h urinary albumin excretion (UAE) levels are shown. **(D)** The glomerular areas measured in pathological images were markedly increased in db/db mice (also see the periodic acid–Schiff staining images below). Data are presented as mean±standard error (SE), ^*^p<0.05, ^**^p<0.01, and ^***^p<0.001 (*t*-test). **(E)** Representative pathological images are shown. (Upper) Periodic acid–Schiff staining images, bar=50μm. (Down) Electron microscopy images with a 4,200-fold magnification. The extensive fusion of foot processes (red arrows) in db/db mice was noticeable.

Body weight and overnight fasting abdominal blood glucose (FBG) levels were significantly higher in db/db mice than in db/m mice during the observation period (Figures 2A,B). In addition, 24-h urine albumin excretion (UAE) levels were significantly higher in db/db mice at 12 and 24weeks of age with glomerulomegaly compared with db/m mice (Figures 2C,D), revealing renal damage. Representative histopathological and electron micrographic data are shown (Figure 2E). PAS staining images (Figure 2E; upper) indicated prominent glomeruli hypertrophy accompanied by mesangial expansion in db/db mice, while the fusion of the podocyte foot processes (red arrows) was noticeable according to electron microscopy images (Figure 2E down) in db/db mice.

Serum levels of renal function and lipid indicators are listed in Table 1. The levels of serum glycated serum protein (GSP), serum uric acid (UA), triglyceride (TG), cholesterol (CHO), and high-density lipoprotein (HDL) were significantly higher in db/db mice compared with db/m mice, indicating substantial glucose and lipid dysregulation induced by diabetes. In addition, the level of malondialdehyde (MDA), one of the by-products of lipid peroxidase and thus an indicator of the extent of lipid peroxidation, was also significantly increased in db/db mice. However, there was no clear difference between the two groups in the levels of serum creatinine (SCr), possibly because of the limitations of specificity in the detection of type 2 DKD in this mouse model (Giralt-Lopez et al., 2020). Together, these pathological and biochemical parameter alternations indicate the progression of type 2 DKD (Brosius et al., 2009).

**Table 1:** Serum biochemical parameters between db/m mice and db/db mice.

Parameter	db/m(*n* =7)	db/db(*n* =6)
MDA (nmol/ml)	3.82±0.41	9.40±3.24^*^
BUN (mg/dl)	27.15±4.89	24.68±4.02
SCr (μmol/L)	24.55±7.97	22.20±8.87
UA (μmol/L)	125.49±25.61	204.99±34.45^*^
GSP (mmol/L)	2.57±0.62	4.61±0.31^*^
TG (mmol/L)	0.73±0.18	1.84±0.66^*^
CHO (mmol/L)	1.80±0.36	3.39±0.47^*^
HDL (mmol/L)	1.20±0.17	2.30±0.06^*^
LDL (mmol/L)	0.22±0.06	0.32±0.16

*Data are presented as mean±standard error (SE). MDA, malondialdehyde; BUN, Blood Urea Nitrogen; SCr, Serum Creatinine; UA, Uric Acid; GSP, Glycated Serum Protein; TG, Triglyceride; CHO, Cholesterol; HDL, High-density Lipoprotein; and LDL, Low-density Lipoprotein. Compared with db/m group*.

* *p<0.001 (t-test)*.

### Histology-Specific Molecular Characterization of Metabolites

**Figure 3:**
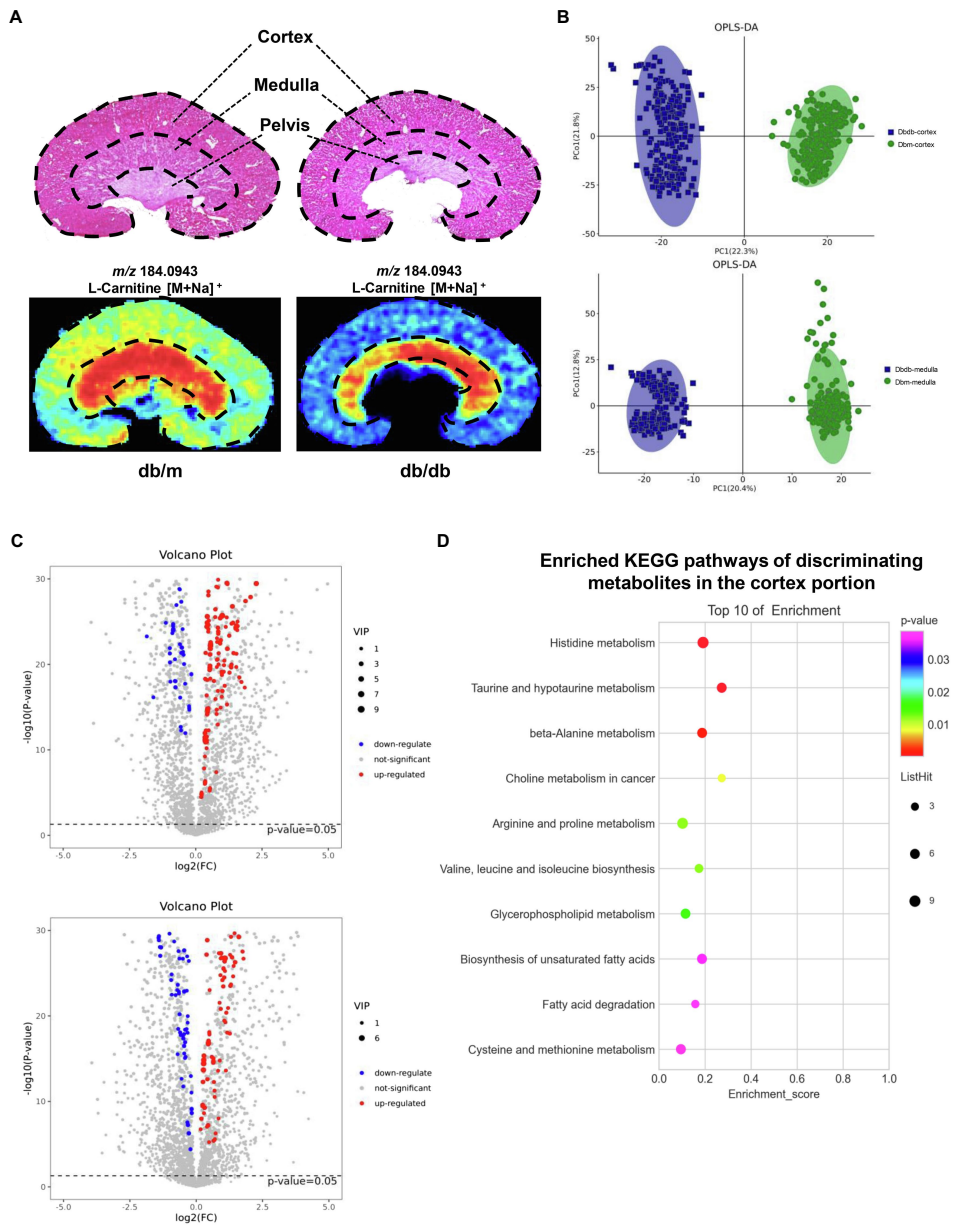
AFADESI–MSI analysis between kidneys of db/m (*n*=2) and db/db mice (*n*=2). **(A)** H&E staining with the structure diagram of mice kidney (upper) and MSI of a representative metabolite(l-carnitine) in db/m and db/db kidney are shown. **(B)** OPLS–DA plots of the metabolites detected in MSI negative ion mode in the cortex (upper) and medulla (down) for kidneys of db/m and db/db mice. **(C)** Volcano plots showing the discriminating metabolites in the cortex (upper) and medulla (down) of db/db vs. db/m mice in negative ion mode. **(D)** The bubble plot showing the enriched KEGG pathways of discriminating metabolites in the cortex portion between the kidneys of db/db and db/m mice.

Kidney tissues consist of the cortex, medulla, and pelvis, all of which have distinctive main functions. In this study, we focused on the renal cortex and medulla portions. H&E staining was implemented on frozen tissue sections to distinguish histological features by a renal pathologist, and AFADESI–MSI experiments were performed in both the positive and negative ion mode on the adjacent tissue sections to acquire molecule profiling of the metabolites. Figure 3A illustrates one representative histology-specific MSI in the positive ion mode, in which the distribution of L-carnitine matches well with its histological features. Moreover, as depicted in the figure, the ion intensities were significantly different between db/m mice and db/db mice among the cortex and medulla, respectively. In total, 509 and 507 biologically informative peaks were observed in the renal cortex and medulla, respectively, for the negative ion mode, whereas in the positive ion mode, 724 and 728 peaks were probed in the renal cortex and medulla, respectively (not shown). In order to explore the overall differentiation of metabolic molecules in the DKD, OPLS–DA was used to choose histology-specific metabolic biomarkers based on the MSI pixel point in the negative ion mode (Figure 3B) and positive ion mode (Supplementary Figure 1A). As a result, both the renal cortex and medulla parts, respectively, could be clearly distinguished between db/db and db/m mice, indicating striking separations in the metabolite profiling of not only the cortex but also the medullary portion induced by T2DM. Next, we selected discriminating metabolites with clear identification potential from the two groups, using a variable importance in projection threshold (VIP)≥1.0 generated by OPLS–DA, and *p*<0.05 according to one-way analysis of variance (ANOVA). Volcano plots were drawn to visualize the dysregulated metabolites in the renal cortex and medulla between the two groups (Figure 3C for negative ion mode; Supplementary Figure 1B for positive ion mode). To further identify significantly perturbated metabolic pathways that may be involved in DKD, we performed metabolic pathway matching analysis by importing the discriminating metabolites into the kyoto encyclopedia of genes and genomes (KEGG) database (Sun et al., 2019; Chen et al., 2021b). We observed that taurine and hypotaurine metabolism, arginine and proline metabolism, histidine metabolism, biosynthesis of unsaturated fatty acids, and fatty acid degradation pathways were significantly dysregulated in DKD (Figure 3D for cortex portion, Supplementary Figure 1C for medulla portion). In total, the *in situ* molecular profiles of the kidneys in a T2DM mouse model based on a histology-specific analysis were first presented, suggesting that several metabolic pathways were remodeled in both the cortex and the medulla portion of a diabetic kidney.

### Disturbance of Crucial Metabolic Pathways Associated With DKD *in situ*

**Figure 4:**
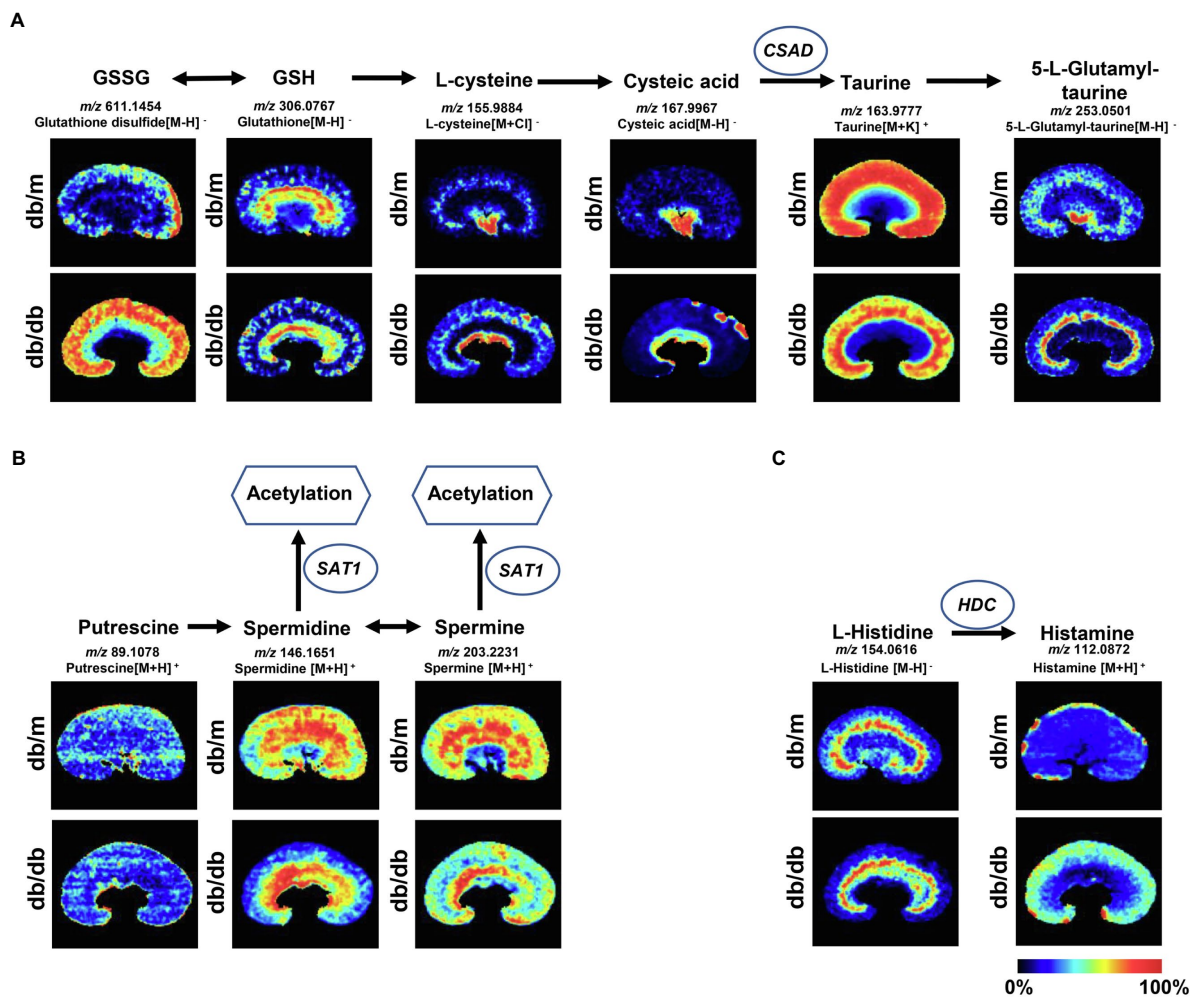
Mass spectrometry images of discriminating metabolites involved in the disturbance of metabolic pathways associated with DKD. Spatial distribution and changes of metabolites involved in **(A)** taurine and hypotaurine metabolic pathway, **(B)** arginine and proline metabolism, and **(C)** histidine metabolic pathway. The blue ellipses represent rate-limiting enzyme that regulate specific metabolic pathways. GSSG, Glutathione disulfide; GSH, glutathione; CSAD, sulfinoalanine decarboxylase; HDC, histidine decarboxylase; and SAT1, spermidine/spermine N1-acetyl transferase 1.

**Figure 5:**
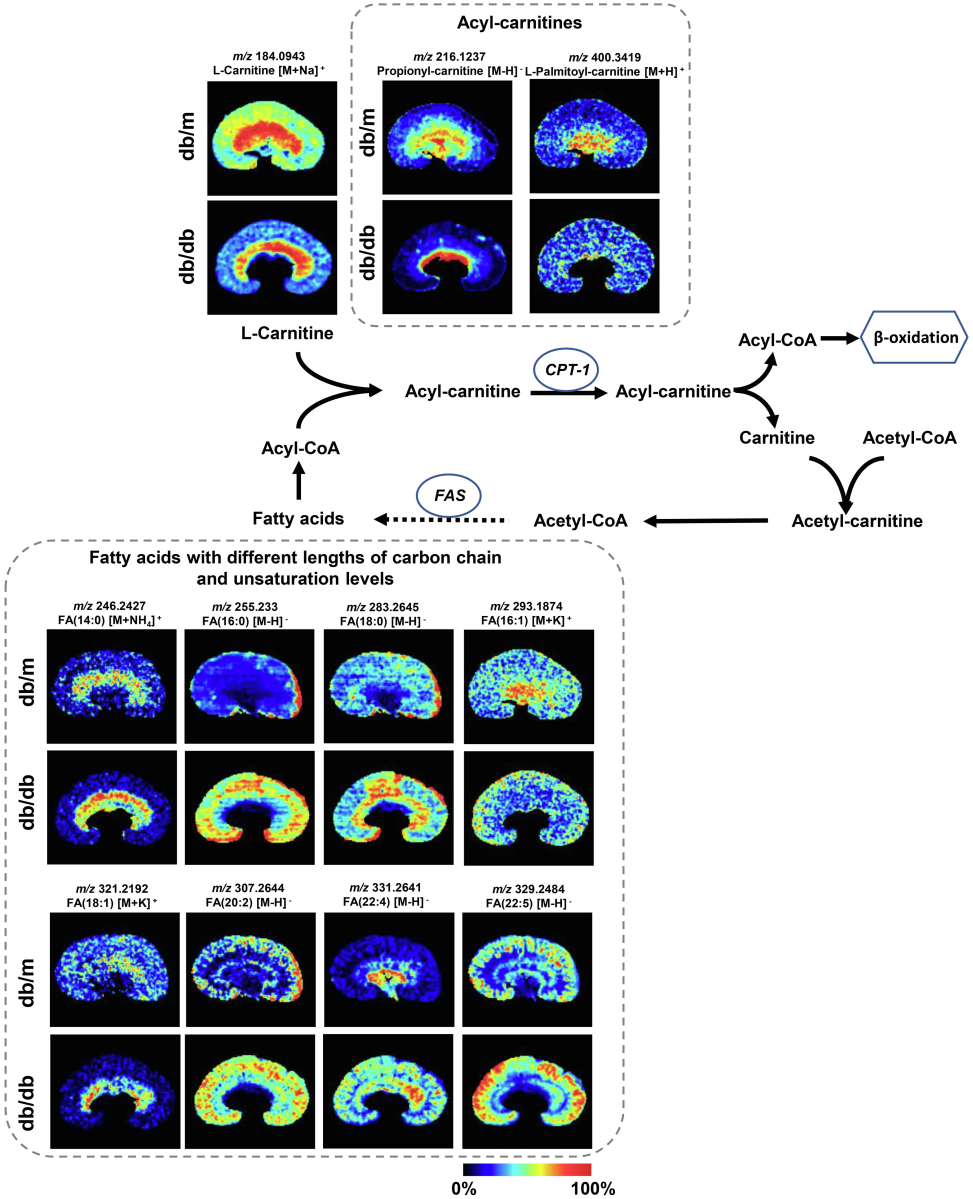
Mass spectrometry images of discriminating metabolites involved in the disturbance of fatty acid degradation pathway and biosynthesis of fatty acids pathway. The blue ellipses represent rate-limiting enzymes that regulate certain metabolic pathways. FA, fatty acid; CPT1, carnitine O-palmitoyl-transferase 1; and FAS, fatty acid synthase.

To further investigate the disturbance of these metabolic pathways induced by DKD, 22 discriminating metabolites with a mass accuracy of <5ppm involved in these metabolic pathways were tentatively selected (Table 2; Figures 4, 5). The distribution of these metabolites clearly matches well with renal histology. In db/m mice, many of the detected metabolites were distributed throughout both the renal cortex and medulla, such as spermidine, spermine (Figure 4B), and L-carnitine (Figure 5). However, several were located within different parts of the kidney. In particular, glutathione disulfide (GSSG) was mainly detected within the cortex (Figure 4A), while L-histidine (Figure 4C) was abundant along the renal corticomedullary junction. Based on histology, many of these metabolites are specifically distributed in the kidney tissues, suggesting that they may perform different function in different regions.

**Table 2:** Metabolites tentatively identified in the AFADESI–MSI with renal distribution information in db/m and db/db mice.

Metabolite identification	Adduct	*m/z*	HMDB	KEGG compound ID	Tissue distribution	
					db/m	db/db
Glutathione disulfide	[M-H]^−^	611.14543	HMDB0003337	C00127	C	C(↑), M(↑)
Glutathione	[M-H]^−^	306.07672	HMDB0000125	C00051	C, M	C, M(↓)
L-Cysteine	[M+Cl]^−^	155.98843	HMDB0000574	C00097	CMJ	CMJ(↑)
Cysteic acid	[M-H]^−^	167.99666	HMDB0002757	C00506	C	C
Taurine	[M+K]^+^	163.97771	HMDB0000251	C00245	C, M	C(↓), M(↓)
5-L-Glutamyl-taurine	[M-H]^−^	253.0501	HMDB0004195	C05844	CMJ	CMJ(↑)
Putrescine	[M+H]^+^	89.107835	HMDB0001414	C00134	C	C (↑)
Spermidine	[M+H]^+^	146.16513	HMDB0001257	C00315	C, M	C(↓), M(↓)
Spermine	[M+H]^+^	203.22313	HMDB0001256	C00750	C, M	C(↓), M(↓)
L-Histidine	[M-H]^−^	154.06156	HMDB0000177	C00135	CMJ	CMJ
Histamine	[M+H]^+^	112.08721	HMDB0000870	C00388	C	C (↑)
L-Carnitine	[M+Na]^+^	184.09431	HMDB0000062	C00318	C, M	C(↓), M(↓)
Propionyl-carnitine	[M-H]^−^	216.12373	HMDB0000824	C03017	C, M	M(↓)
L-Palmitoyl-carnitine	[M+H]^+^	400.3419	HMDB0000222	C02990	C, M	C, M(↓)
FA(14:0)	[M+NH_4_]^+^	246.2427	HMDB0000806	C06424	M	CMJ(↑), M(↑)
FA(16:0)	[M-H]^−^	255.23295	HMDB0000220	C00249	C	C(↑), CMJ(↑), M(↑)
FA(18:0)	[M-H]^−^	283.26445	HMDB0000827	C01530	C, M	C(↑), CMJ(↑), M(↑)
FA(16:1)	[M+K]^+^	293.18744	HMDB0003229	C08362	C, M	C, M(↓)
FA(18:1)	[M+K]^+^	321.21922	HMDB0003231	C08367	C, M	C(↓), M
FA(20:2)	[M-H]^−^	307.26436	HMDB0005060	C16525	C	C(↑), M(↑)
FA(22:4)	[M-H]^−^	331.26413	HMDB0002226	C16527	M	C(↑), M(↑)
FA(22:5)	[M-H]^−^	329.24845	HMDB0006528	C16513	C, M	C(↑), M(↑)

*C, cortex; CMJ, corticomedullary junction; M, medulla; and FA, fatty acid. (↑) Indicates significant increase in local abundance; (↓) denotes significant decrease in local abundance*.

The metabolites were evenly distributed in their particular regions of the kidney. However, compared with db/m mice, there were significant alternations in the diabetic kidney. Notably, diabetes-induced kidney metabolic reprogramming not only occurs in the cortex portion (which contains most of the glomerulus and proximal renal tubules that serve crucial filtration, reabsorption, and endocrine functions), but also in the medulla (which mainly performs reabsorption function). Compared with db/m mice, the relative abundances of GSSG were much greater in the renal cortex of db/db mice. Meanwhile, clear ion intensities are noted in the renal medulla of db/db mice, whereas very little was detected in the same portion of db/m mice (Figure 4A). In addition, taurine, spermidine, and spermine were observed at lower relative abundances in both the renal cortex and medulla of db/db mice (Figures 4A,B). In the renal cortex, diabetic mice presented relatively higher intensities of histamine (Figure 4C); however, the relative ion intensities of L-palmitoyl-carnitine and propionyl-carnitine (Figure 5) were lower in the diabetic renal medulla. Moreover, a list of fatty acids with different lengths of carbon chain and unsaturation levels was observed in our MSI study (Figure 5), many of which showed different distribution and abundance characteristics. Molecular distributions of saturated long-chain fatty acids, including FA (14:0), FA (16:0), and FA (18:0), presented higher intensities in the cortex and/or medulla of db/db mice, suggesting accumulation in these important regions when exposures to DKD. It was also observed that long-chain polyunsaturated fatty acids, including FA (20:2), FA (22:4), and FA (22:5), were significantly upregulated in both the cortex and the medulla portions of the diabetic kidney. However, two monounsaturated fatty acids detected in the present study showed reduced relative ion intensities in the kidney of db/db mice. The abundance of FA (16:1) and FA (18:1) was significantly reduced in the renal medulla and cortex, respectively. In summary, we discovered several significant metabolic pathway transformations *in situ* induced by DKD.

### Metabolome Analysis of Renal Tissue

**Figure 6:**
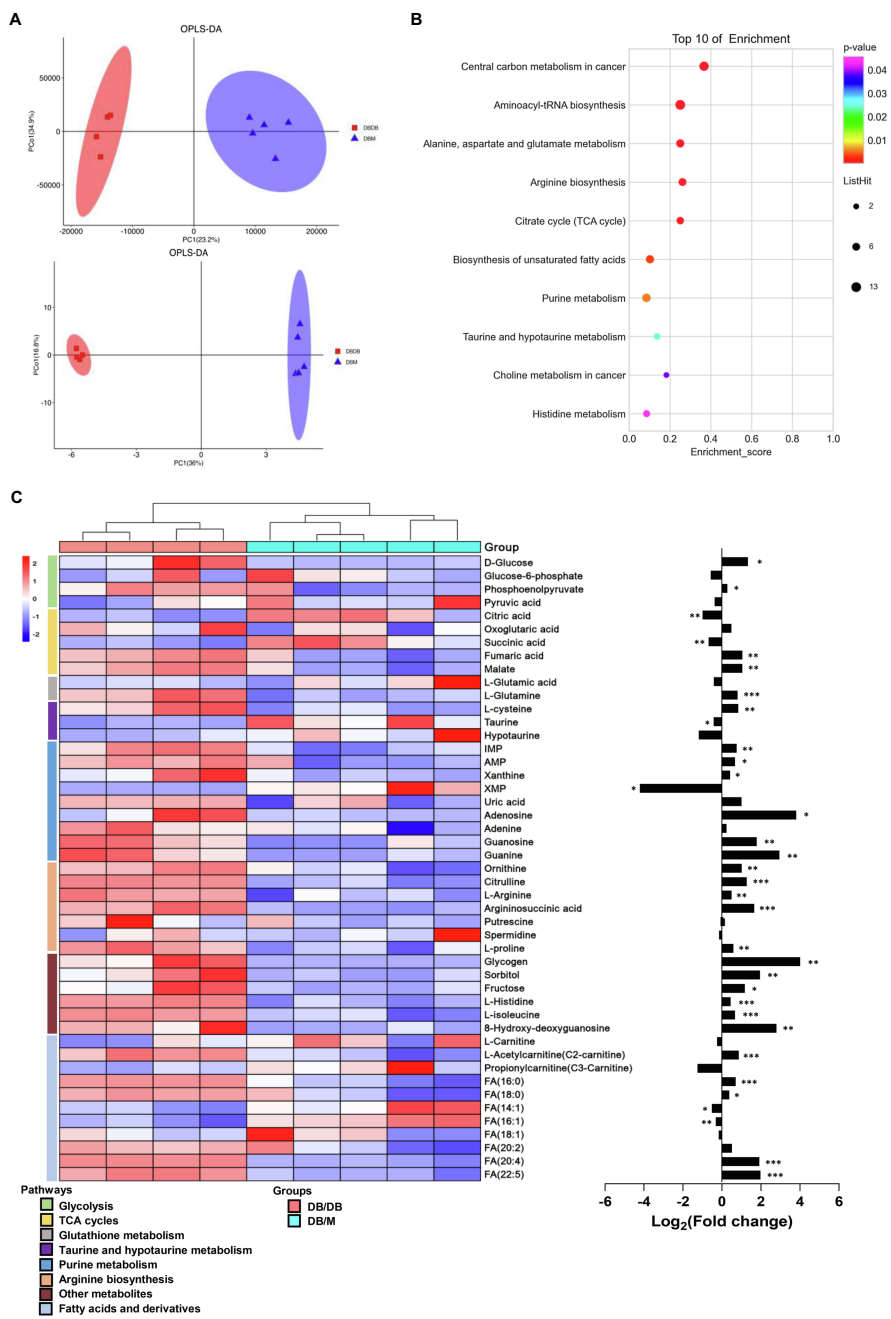
Integrated metabolomics analysis between kidneys of db/m (*n*=5) and db/db (*n*=4) mice. **(A)** OPLS–DA plots of the metabolites detected in kidneys of db/m and db/db mice in GC–MS (upper) and UPLC–MS (down). **(B)** The bubble plot showing the integrated of GC–MS and UPLC–MS enriched in KEGG pathways of metabolites with VIP score≥1.0 between kidneys of the two groups. **(C)** The heat map (left) illustrating the levels of metabolites in kidneys of db/m and db/db mice, each row represents one metabolite, each column represents one sample. Bar graphs (right) showing fold changes converted to logarithms in db/db mice compared to db/m mice. ^*^p<0.05, ^**^p<0.01, and ^***^p<0.001 difference between db/m and db/db mice (*t*-test).

We also examined the whole renal tissue using traditional metabolic methods to detect the total amounts and types of metabolites. Here, we performed integrated metabolomics studies using GC–MS and UPLC–MS techniques together to improve the accuracy, integrity, and sensitivity of the identification of the phenotype-related metabolites (Zeki et al., 2020). A total of 1,544 peaks were detected by GC–MS and UPLC–MS (342 and 1,202 peaks were obtained from GC–MS and UPLC–MS, respectively, data not shown) from renal tissue. Scoring plots generated from OPLS–DA models presented a clear separation between db/db mice and db/m mice (Figure 6A). Next, we linked the metabolites with VIP score≥1.0 to metabolic pathways using the KEGG database (Figure 6B). Differences in taurine and hypotaurine metabolism, arginine metabolism pathways, histidine metabolism, and biosynthesis of unsaturated fatty acids were also observed, as in our MSI results. Moreover, purine metabolism and glucose metabolism processes, such as citrate cycle (TCA cycle), were enriched. A heat map for each metabolite involved in the metabolic pathways of interest and a bar graph for the fold changes of the metabolites of db/db compared with db/m mice is presented (Figure 6C). Interestingly, in the MSI experiments, the expression of putrescine, spermidine, L-carnitine, propionyl-carnitine, FA (18:1), and FA (20:2) was significantly different between the two groups, resulting in no statistical differences in metabolome analysis between db/db and db/m mice (although the trends were consistent to MSI data). This confirms that traditional metabolomics approaches using bulk tissue measurements result in the average measurements, which may lose key information from different tissue areas (Andersen et al., 2021).

### Visualization of Metabolome Data on the Associated Metabolic Pathway Map

**Figure 7:**
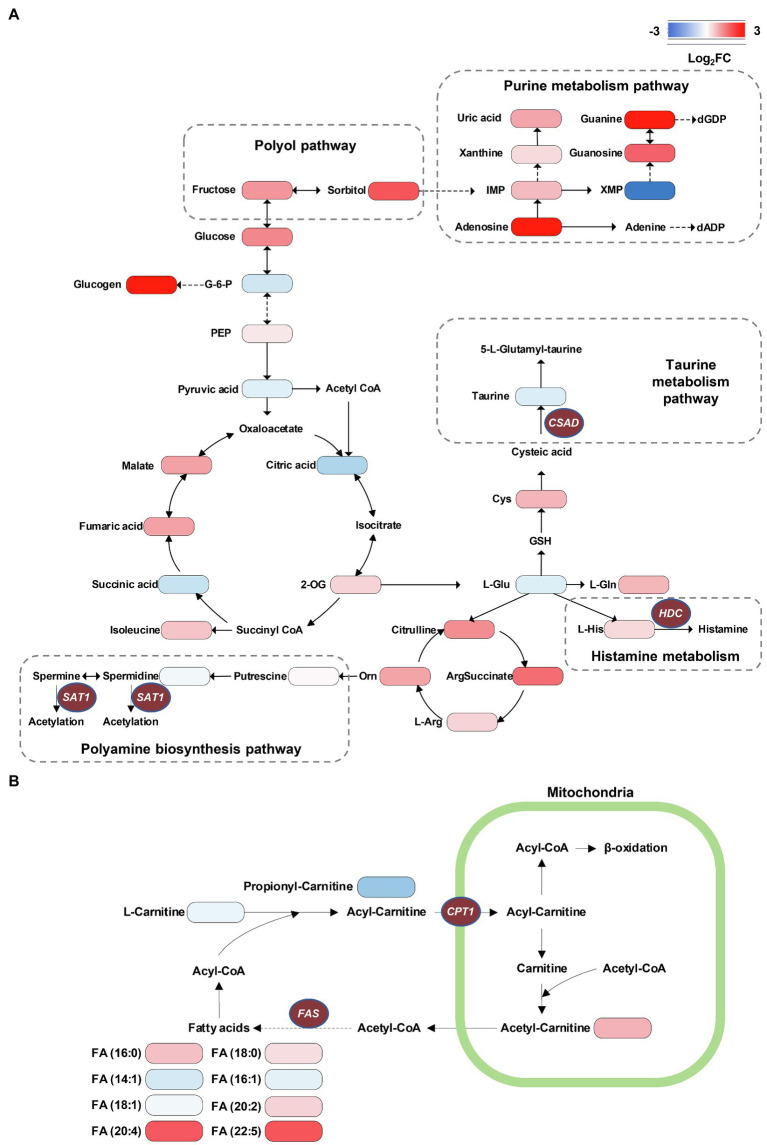
Visualization of metabolome data on the energy metabolic pathway map. **(A)** Accumulation of TCA cycle metabolites, taurine consumption, spermidine consumption, accumulation of polyol pathway metabolites, and accumulation of purine metabolism pathway metabolites can be recognized in db/db compared with db/m mice. **(B)** The alternations of long-chain saturated and polyunsaturated fatty acids are presented. The brown ellipses represent rate-limiting enzymes that regulate specific metabolic pathways. G-6-P, glucose-6-phosphate; PEP, phosphoenolpyruvate; 2-OG, oxoglutarate; IMP, inosinic acid; XMP, xanthylic acid; dGDP, 2'-deoxyguanosine 5'-diphosphate; dADP, 2'-deoxyadenosine 5'-diphosphate; L-Gln, L-glutamine; L-Glu, L-glutamate; GSH, glutathione; Cys, cysteine; L-Arg, L-arginine; Orn, ornithine; HDC, histidine decarboxylase; CPT1, carnitine O-palmitoyl-transferase 1; FAS, fatty acid synthase; SAT1, Spermidine/spermine N1-acetyl transferase 1; and CSAD, sulfinoalanine decarboxylase.

Investigation into the metabolic pathway alternations involved in db/db mice was performed using the KEGG database (Figure 7). We verified that several glycolysis and TCA cycle intermediates were accumulated in the diabetic kidney, such as phosphoenolpyruvate (PEP), fumaric acid, and malate, suggesting an upregulation of glycolysis and the TCA cycle due to the excessive glucose inflow in DKD (Figure 7A). This finding is compatible with previous studies (Sas et al., 2016; Hasegawa et al., 2020). Moreover, several purine nucleoside concentrations were upregulated in db/db mice, indicating the upregulation of the purine metabolism. In contrast, the lower level of taurine indicated a reduced taurine synthetic metabolism (Figure 7A). The accumulation of long-chain saturated and polyunsaturated fatty acids was also shown (Figure 7B). Together, and consistent with our MSI data, the metabolic pathway alternations were confirmed according to metabolomic pathway analysis using the entire tissue bulk.

### Validation of Key Metabolic Enzymes in Diabetic-Associated Metabolic Pathways *in situ*

**Figure 8:**
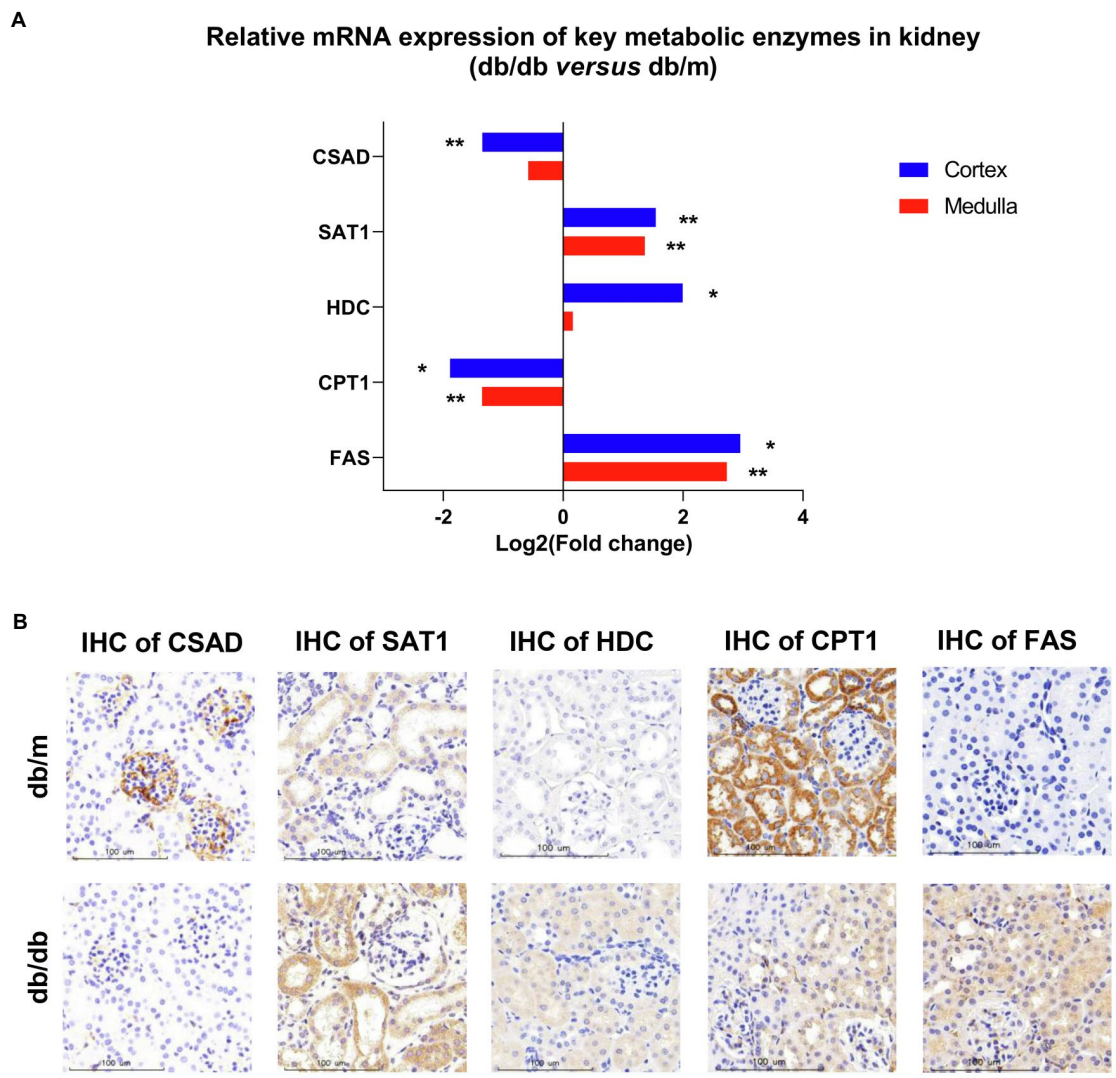
The validation of real-time PCR and specific immunohistochemistry (IHC) for different rate-limiting enzymes in specific metabolic pathways between db/m (*n*=7) and db/db (*n*=6) mice kidneys. **(A)** Levels of mRNA expression in renal cortex and medulla according to real-time PCR are shown. The data using fold changes converted to logarithms of db/db vs. db/m mice. ^*^p<0.05; ^**^p<0.01 (*t*-test). **(B)** Representative IHC of rate-limiting enzymes including CSAD, SAT1, HDC, CPT1, and FAS in the kidneys between db/m and db/db mice are shown. bar=100μm.

As crucial links in metabolic pathways, the rate-limiting enzymes control the rate and direction of metabolic processes. In the present study, we observed several remodeling metabolic pathways occurring in DKD, including taurine and hypotaurine metabolism, arginine and proline metabolism, histidine metabolism, biosynthesis of unsaturated fatty acids, and fatty acid degradation pathway. We next detected the expression level of rate-limiting enzymes in specific metabolic pathways. We employed real-time PCR analysis to detect the mRNA expression levels of rate-limiting enzymes involved in these metabolic pathways (Figure 8A). We further examined the *in situ* expression levels of these enzymes by performing IHC on kidney tissues (Figure 8B). The detailed rate-limiting enzymes are presented in Supplementary Table S3. Specifically, sulfinoalanine decarboxylase (CSAD) catalyzes the biosynthesis of taurine. Compared with db/m mice, there was a clear decrease in mRNA expression of CSAD in the renal cortex of db/db mice, but no statistical difference in medulla portions (*p*=0.06). In addition, IHC analysis indicated that CSAD was mainly expressed in the glomeruli with a small amount in the tubules in db/m mice, although there was a significant decrease in both the glomeruli and tubules of db/db mice. Carnitine O-palmitoyl-transferase 1 (CPT1) is the rate-limiting enzyme of fatty acid oxidation (FAO), allowing long-chain fatty acids shuttling from the mitochondrial outer membranes into the mitochondrial matrix. This was mainly located in the tubules of db/m mice and reduced in db/db mice according to IHC data. Spermidine/spermine N1-acetyl transferase 1 (SAT1) catalyzes the acetylation of spermidine and spermine to deplete them, histidine decarboxylase (HDC) induces the decarboxylation of histidine to form histamine, and fatty acid synthase (FAS) stimulates the formation of long-chain fatty acids. In db/db mice, the mRNA expression levels of SAT1 and FAS were substantially upregulated in both the cortex and medulla portions, whereas the significant mRNA accumulation of HDC was detected only in the cortex. Further, IHC staining of SAT1 indicated an increasing trend in tubulars in db/db mice kidney, and the subsequent IHC assay of HDC and FAS showed that they were upregulated in both glomeruli and tubulars of db/db mice. Together, using these approaches, we verified that taurine biosynthesis, arginine and proline metabolism, histidine metabolism, unsaturated fatty acid biosynthesis, and fatty acid degradation pathways were altered in DKD.

## Discussion

The major aim of the present study was to identify the differential metabolic molecular profiles *in situ* as well as the associated alternations of metabolic pathways and specific metabolic enzymes that are involved in type 2 DKD. By using a high-coverage ambient MSI technology, we identified 22 significant differential metabolites *in situ* that showed a good spatial match with histology. Subsequently, a metabolic pathway matching analysis was performed to further explore the underlying biological functions of these metabolites. This analysis suggested that (a) taurine metabolism, (b) arginine and proline metabolism, (c) histidine metabolism, (d) biosynthesis of unsaturated fatty acids, and (e) fatty acid degradation pathways were remodeled, which was associated with DKD. Further, we conducted a traditional metabolomics analysis to detect the total amount and type of metabolites in whole kidney tissues using GC–MS and UPLC–MS techniques in combination, before a metabolic network was established. This was compatible with our MSI data. Next, to validate the expression profiles of the potential enzymes of the metabolic pathways above, real-time PCR and IHC were performed to detect mRNA and the spatial expression levels of specific enzymes, respectively. It should be noted that the remarkably dysregulated DKD-associated metabolic pathways have numerous rate-limiting enzymes; in this study, we focused on key enzymes with directly up- or down-stream relationship with the significantly altered metabolites in certain pathways. In particular, db/db mice had decreased relative abundances of CSAD and CPT1, and increased relative levels of SAT1, HDC, and FAS in the kidney tissue. The significantly altered metabolites and metabolic pathways are discussed in detail below.

### Disturbance on Taurine Metabolism

It is well known that, as an essential antioxidant, taurine (a sulfur-containing amino acid) participates in the prevention of oxidant generation (Schaffer et al., 2009) and lipid peroxidation (Parvez et al., 2008), and has increasingly captured attention due to its inhibitory effects of DKD (Koh et al., 2014; Zhang et al., 2020b). Moreover, taurine may protect mitochondria due to a reduction in mitochondrial reactive oxygen species (ROS) production and a partial recovery of mitochondrial Mn-superoxide dismutase (Chang et al., 2004). The MSI data based on histology in the present study indicate that the ion intensity of taurine in the renal cortex and medulla of db/db mice is significantly lower than that in db/m mice, as also confirmed by metabolome data. Based on the taurine metabolic pathway, CSAD is a crucial rate-limiting enzyme for the biosynthesis of taurine (Park et al., 2017), and its expression plays a pivotal role in the pathogenesis of T2DM in β-cells (Chen et al., 2021a). We assume that the decreased levels of taurine in the diabetic kidney may be attributed to the downregulated expression of CSAD. Real-time PCR and IHC staining data were consistent with our assumption. Interestingly, in db/m mice, the IHC analysis showed that CSAD was mainly expressed in the glomeruli with a small amount in the proximal tubules, which is consistent with the high abundance of taurine in the cortex portions according to our MSI data. To our knowledge, this is the first time that the molecular profiles of taurine have been detected with MSI in type-2 DKD mouse kidney tissue. The decreased abundance of taurine in the diabetic kidney is probably due to the low expression of CSAD.

### Perturbation on Arginine and Proline Metabolism

In mammals, arginine can be catalyzed to polyamines, including putrescine, spermidine, and spermine. There is growing evidence suggesting that the dysregulation of polyamine metabolism can cause changes in high glucose-induced energy perturbation, including streptozotocin (STZ)-induced diabetic cardiomyopathy (Wang et al., 2020a), type 1 DKD (Zhang et al., 2021), and retinopathy (Liu et al., 2020), thus making it a promising target for therapeutic intervention. It has been reported that, as ROS scavengers, spermine and spermidine can protect DNA from free radical attacks, regulating cell proliferation, differentiation, and apoptosis (Pegg, 2009; Wang et al., 2020a). SAT1, which catalyzes the acetylation of spermidine and spermine, is the rate-limiting enzyme in the catabolism of polyamine, and overexpression of SAT1 results in an overall depletion of spermidine and spermine (Mandal et al., 2013). In this study, the abundance of spermidine and spermine was dramatically decreased in the diabetic kidney compared with db/m mice, especially in the cortex portions, although the metabolome data showed no significant difference in spermidine between the two groups, which may be due to the whole kidney tissue bulk analysis. According to these results, we speculated that the downregulated expression of spermidine and spermine may be the result of ascending SAT1. The subsequent real-time PCR and IHC validation were then performed to evaluate the mRNA and protein expression of SAT1 in the tissue, respectively. As predicted, db/db mice showed a higher mRNA expression of SAT1 in both the renal cortex and medulla, and also a stronger level of SAT1 expression *in situ* compared with db/m mice. These findings suggest a potential role of SAT1 in the development of type 2 DKD, as reported in a previous study (Zhang et al., 2021).

### Disturbance on Histidine Metabolism

Histamine, decarboxylated from histidine induced by HDC, has four cognate G protein-coupled receptors, namely, H1R to H4R. Histamine exerts pro-fibrotic and pro-inflammatory effects through these receptors during the development of DKD, wherein the inhibition of H1R can maintain the integrated morphology of podocytes (Veglia et al., 2016), whereas inhibition of H4R can attenuate the reabsorptive dysfunction in proximal tubules in STZ-induced DKD (Pini et al., 2018). In particular, type-1 DKD rats showed significantly higher HDC activity in the kidney, and thus an increased abundance of histamine (Gill et al., 1990). Our MSI data suggested that histamine biosynthesis was severely upregulated in the renal cortex of db/db mice, and the subsequent real-time PCR performed to test the mRNA expression of HDC also showed a significantly higher level in the cortex portions. Interestingly, mast cells, which are well known as the major producer of histamine in tissues, could be scarcely found in the present study according to histological examinations. The IHC staining demonstrated an increased tendency of HDC mainly in the proximal tubules and to a lower extent in the glomeruli in db/db mice, suggesting the existence of a local histamine pool of intrinsic renal cells induced by type 2 DKD (Pini et al., 2019).

### Dysregulation of Fatty Acid Metabolism

It is known that the kidney needs a large energy supply for its filtering and reabsorption functions. Renal tubules, especially the proximal convoluted tubule, thick ascending loop, and distal convoluted tubule that are mainly located at the outer medulla and corticomedullary junction, contain most of the renal mitochondria, in which ATP generation mainly depends on FAO (Bhargava and Schnellmann, 2017; Forbes and Thorburn, 2018). Dysfunctional FAO has been found in the development of DKD in several studies, although the mechanism remains controversial (Sas et al., 2016; Sha et al., 2020). On the other hand, the accumulation of long-chain fatty acids induced by DKD may negatively control FAO by creating a lipotoxicity environment inside the proximal tubules (Ruggiero et al., 2014; Simon and Hertig, 2015). By binding to albumin for the formation of fatty acid-binding proteins (FABPs), fatty acids can be filtered by glomeruli and reabsorbed by tubules (Bhargava and Schnellmann, 2017). As albuminuria excretion increases during the development of DKD, the filtration of FABPs is overloaded, resulting in a significantly increased reabsorption status, which induces severe damage in the tubules (Cobbs et al., 2018). Of note, according to our MSI and metabolome data, fatty acids with different lengths of carbon chains and numbers of double bonds showed differing abundance and location between db/db and db/m. The concentration of long-chain fatty acids, including both saturated [e.g., FA (16:0), FA (18:0)] and polyunsaturated [e.g., FA (20:4), FA (22:5)] acids, was significantly increased in the diabetic kidney, whereas monounsaturated fatty acids [e.g., FA (16:1), FA (18:1)] were less enriched compared with db/m mice. Similarly, glomerular and tubulointerstitial lipid accumulation has been found in both type 2 DKD patients (Herman-Edelstein et al., 2014) and db/db mice (Wang et al., 2005), and we further identified complex fatty acid composition on the spatial level to validate the accumulation. The higher abundance of FA (16:0) may induce inflammation and apoptosis in the renal proximal tubular cell (Soumura et al., 2010) and mitochondrial oxidative stress in podocytes (Xu et al., 2015), and the kidney is easily damaged by ROS due to the high level of polyunsaturated fatty acids (Zhang et al., 2020a). On the other hand, the administration of monounsaturated fatty acid FA (16:1) to KKAy mice (a T2DM model with low insulin sensitivity) can reduce fasting glycemia and insulin resistance in parallel with reduced relative mRNA expression of FAS (Yang et al., 2011). These results indicate the latent role of various types of fatty acids in progression of DKD, which may be involved in the progression of DKD.

Another significant finding for fatty acid metabolism in DKD is the dysregulation of FAO. As previously mentioned, proximal tubules utilize non-esterified fatty acids [e.g., FA (16:0)] via binding to carnitine, resulting in the formation of acyl-carnitines (e.g., palmitoyl-carnitine). Then, acyl-carnitines can be translocated by CPT1 into mitochondria for FAO to produce the main source of energy. In DKD, the dysfunction of FAO has been documented in several studies, although the results differ, partly because of the different DKD stages at which these studies were observed (Li et al., 2013; Sas et al., 2016; Afshinnia et al., 2019). In general, FAO fluxes in the renal tissues were higher in the early stage of DKD, with significant reduction in advanced DKD (Hasegawa and Inagi, 2021). According to the present study, the concentration of two acyl-carnitines was decreased in the diabetic kidney medulla. It has been reported that palmitoyl-carnitine can serve as a marker of FAO rate, and its low level may be attributed to the impaired β-oxidation in the diabetic kidney due to the decreased CPT1 (Bouchouirab et al., 2018; Afshinnia et al., 2019). In addition, the decreased abundance of propionyl-carnitine, a product of mitochondrial branched-chain amino acid (BCAAs) catabolism also reported to be an antioxidant agent, may also be the consequence of impaired FAO (Adams et al., 2009; Scioli et al., 2014).

Considering the above findings, we surmised that CPT1 would be weaker in the diabetic kidney, since it was demonstrated that the lower expression of CPT1 was related to tubulointerstitial fibrosis from DKD patients (Li et al., 2017). The real-time PCR and IHC data confirmed our assumption. Collectively, the upregulated biosynthesis of several saturated fatty acids by agitating the activity of FAS plus the impaired FAO through the suppressive activity of CPT1 was observed in the dysregulation of fatty acids metabolism, which may provide new insights into the potential lipid-treatment of DKD.

Further research is needed to fully elucidate the underlying mechanism of these metabolic pathways reprogramming. However, alternations of the distribution and abundance of most metabolites highlight the association with mitochondria dysfunction in DKD. The accumulation of fatty acids may damage podocytes and tubulars, which can be associated with ROS formation in mitochondria (Forbes and Thorburn, 2018). On the other hand, insufficient antioxidant capacity is another contributor to mitochondrial dysfunction in DKD. As a fundamental antioxidant, mitochondrial glutathione (GSH) helps to decrease excessive ROS by interacting with the superoxide anions and then being oxidized to GSSG (Lushchak, 2012). However, in db/db mice, it is worth noting that our results indicated a remarkably higher abundance of GSSG in the cortex and medulla accompanied by downregulated GSH in the medullar (Figure 4A), which suggested the high levels of ROS that exceed local antioxidant capacity. Furthermore, our metabolome data also exhibited a clear upregulation of 8-hydroxy-deoxyguanosine (8-OHdG) in db/db mice (Figure 6C), a well-known marker for measuring the effect of oxidative damage to mitochondria DNA (Kakimoto et al., 2002). In general, metabolic pathway reprogramming accompanied by dysfunctional mitochondria could be detrimental to energy production and renal function in DKD, whereas targeted activation or inhibition of the dysregulated metabolic enzymes to regulate the expression of metabolites may have a potential role in metabolism-based therapy. This area warrants further research.

## Conclusion

In summary, this *in situ* metabolic approach based on AFADESI–MSI was developed for the high-throughput investigation for type 2 DKD-associated metabolic and enzymatic alternations. The distribution and histology-specific dysregulations of 22 metabolites were related to taurine and hypotaurine metabolism, arginine and proline metabolism, histidine metabolism, biosynthesis of unsaturated fatty acids, and fatty acid degradation pathways, and 5 anomalously expressed metabolic rate-limiting enzymes were further identified, including CSAD, SAT1, HDC, CPT1, and FAS. The present study offers new insights for understanding the intricate metabolic reprogramming underlying type 2 DKD and could help identify potential therapeutic targets. Furthermore, these findings also highlight that based on AFADESI−MSI, the *in situ* metabolic method was capable for applications in metabolic diseases.

## Data Availability Statement

The original contributions presented in the study are included in the article/**Supplementary Material**, and further inquiries can be directed to the corresponding author.

## Ethics Statement

The animal study was reviewed and approved by the Animal Care and Use Committee of Shanghai Jiao Tong University School of Medicine affiliated Tongren Hospital.

## Author Contributions

WX conceived and supervised the study and acquired funding. BL, WY, and ZL performed the AFADESA-MSI and metabolomics analysis and data analysis. BL, LY, and CS performed the animal study and/or contributed to the materials. BL and WY performed the validation experiments. BL prepared the manuscript draft. BL, XX, LF, and WX revised the manuscript. All authors participated in the discussion and editing of the manuscript.

## Funding

This work was supported by the National Natural Science Foundation of China (Grant Nos. 81770718 and 82000687).

## Conflict of Interest

The authors declare that the research was conducted in the absence of any commercial or financial relationships that could be construed as a potential conflict of interest.

## Publisher’s Note

All claims expressed in this article are solely those of the authors and do not necessarily represent those of their affiliated organizations, or those of the publisher, the editors and the reviewers. Any product that may be evaluated in this article, or claim that may be made by its manufacturer, is not guaranteed or endorsed by the publisher.

## Supplementary Material

The Supplementary Material for this article can be found online at: https://www.frontiersin.org/articles/10.3389/fphys.2021. 779683/full#supplementary-material
