# Supplementary material for: In situ Metabolomics of Metabolic Reprogramming Involved in a Mouse Model of Type 2 Diabetic Kidney Disease
Source: Front Physiol. 2021 Nov 30;12:779683. doi: 10.3389/fphys.2021.779683 (PMC8670437; doi:10.3389/fphys.2021.779683)
Supplement: Supplementary file 1 [file Data_Sheet_1.docx]

Supplementary Material

# Supplementary method

## Sample preparation

All kidney tissue samples were stored in −80 ℃ and thawed on ice before analysis. 30 mg accurately weighed samples were transferred into a 1.5-mL Eppendorf tube with 20 μL internal standard (2-chloro-l-phenylalanine in methanol, 0.3 mg/mL) and then mixed with 600 μL extraction solvent with methanol /water (4/1, v/v) were added to each sample. The mixtures were precooled at −20 ℃ for 2 min and then grinded at 60 HZ for 2 min. For GC-MS, 120 μL of chloroform was added to the samples, then vortexed vigorously. Next the mixtures were ultrasonicated in ice water bath for 10 min, rested in −20 ℃ for 1 h. and centrifuged by 13,000 rpm under 4℃ for 15 min. Finally, the supernatant was extracted by using a syringe. For UPLC-MS analysis, 150 μL supernatant for each sample was extracted, passaging through a 0.22-μm filter, and then centrifuged at 15000 g with 4°C for 10 min. For GC-MS analysis, 100 μL supernatant was transferred to a glass sampling vial for vacuum-dry at room temperature before derivatization.

The derivatization for GC-MS analysis was performed by the following steps. First, an 80 μL of methoxylamine hydrochloride (dissolved in pyridine, 15 mg/mL) was subsequently added to each sample, then vortexed vigorously for 2 min and incubated at 37 ℃ for 90 min. Next, 80 μL of bis-(trimethylsilyl)-trifluoroacetamide (BSTFA) contained with 1% TMCS and 20 μL n-hexane were added into each mixture, vortexed for 2 min and then derivatized at 70 ℃ for 60 min. All the samples were rested at room temperature for 30 min before GC-MS analysis.

Quality control (QC) samples were made by mixing aliquots of all samples to generate a pooled sample for UPLC-MS and GC-MS analysis, respectively.

## UPLC–MS analysis

A Nexera UPLC system fitted with Q-Exactive quadrupole-Orbitrap mass spectrometer (Thermo Fisher Scientific, Waltham, MA, USA) equipped with heated electro spray ionization (ESI) source was used to analyze the metabolic profiling in both positive and negative ion modes. UPLC separation was employed on an ACQUITY UPLC HSS T3 (100 mm × 2.1 mm, 1.8 μm) in both positive and negative modes using binary gradient elution system at a flow rate of 0.35 mL/min and column temperature of 45 ℃. The mobile phases were consisted of (A) water (containing 0.1 % formic acid, v/v) and (B) acetonitrile (containing 0.1 % formic acid, v/v) and separation was achieved using the following gradient: 5 % B over 0–2 min, 5–30 % B over 2–4 min, 30–50 % B over 4–8 min,50–80 % B over 8–10 min, 80–100 % B over 10–14 min, the composition was held at 100 % B for 1 min, then 15–15.1 min, 100 % to 5 % B, and 15.1–16 min holding at 5 % B. All the samples were kept at 4℃ during the analysis. The injection volume was 2 μL. The Q-Exactive quadrupole-Orbitrap mass spectrometer was performed in positive and negative ESI mode with ion spray voltage of 3,500 V (+) and 3,000 V (−), a sheath gas flow rate of 40 (+) and 35 (−) arbitrary units, an auxiliary gas flow rate of 10(+) and 8(−) arbitrary units, and a capillary temperature of 320 ℃.

## GC–MS analysis

Each 1 μL derivatized samples was analyzed in splitless mode on a Trace 1310 gas chromatography system coupled to an TSQ 9000 system (Agilent Technologies Inc., CA, USA) carrying a non-polar DB-5MS fused-silica capillary column (30 m × 0.25 mm × 0.25 μm, Agilent J & W Scientific, Folsom, CA, USA). Helium (>99.999%) was used as the carrier gas at a constant flow rate of 1.2 mL / min through the column. The injector temperature was maintained at 300 ℃. The initial oven temperature was 60 ℃, Followed by 8 ℃/min temperature ramp to 125 ℃, then 5 ℃/min to 210 ℃, 10 ℃/min to 270 ℃, 20 ℃/min to 305 ℃ and finally held at 305 ℃ for 5 min. The temperature of MS quadrupole and ion source (electron impact) was set to 280 and 330 ℃, respectively, and the collision energy was 70 eV. Mass spectrometric data was acquired in a full-scan mode in m/z range of 50-500.

## Data processing

The procedure of metabolome data analysis has been described (Sun et al., 2019). Briefly, the obtained UPLC-MS raw data were analyzed by the progenesis QI software (Waters Corporation, Milford, USA) for peak disconvolution, peak alignment, and normalization. Then a data matrix was generated including information of m/z, the retention time, and peak intensity. The ion peaks were identified by progenesis QI Data Processing Software (Waters Corporation, Milford, USA), based human metabolite database (HMDB). Raw MS data from GC-MS were converted and then imported into Mass Spectrometry-Data Independent Analysis software (MS-DIAL version 4) to obtain a data matrix including sample information, retention time, m/z, and peak intensities. All the internal standards and any known pseudo positive peaks (caused by background noise or derivatization procedure) were removed, generating the final dataset for statistical analysis. The ion peaks from GC-MS were annotated in NIST 11 standard mass spectral database Fiehn database.

## References

Sun, C., Li, T., Song, X., Huang, L., Zang, Q., Xu, J., et al. (2019). Spatially resolved metabolomics to discover tumor-associated metabolic alterations. *Proc Natl Acad Sci U S A* 116**,** 52-57.

# Supplementary Figures and Tables

## Supplementary Figure


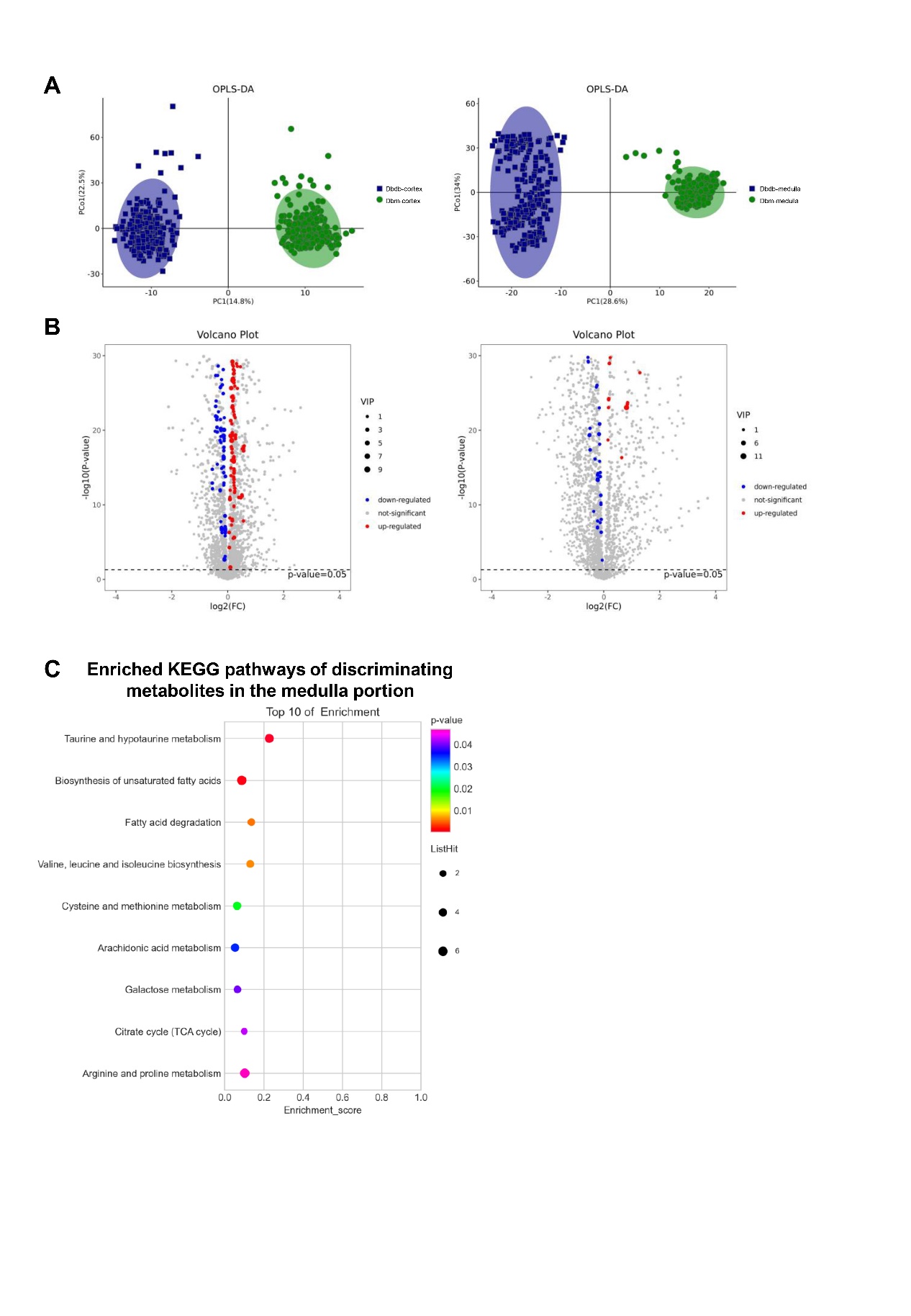


**Supplementary Figure 1.** AFADESI-MSI analysis between kidneys of db/m (n = 2) and db/db mice (n = 2). (A) OPLS-DA plots of the metabolites detected in MSI positive ion mode in the cortex (left) and medulla (right) of kidneys of db/db versus db/m mice. (B) Volcano plots show the discriminating metabolites between the cortex (left) and medulla (right) kidneys of db/m and db/db mice in positive ion mode. (C) The bubble plot shows the enriched KEGG pathways of discriminating metabolites in the medulla portion between the kidneys of db/db versus db/m mice.

## Supplementary Tables

**Supplementary Table S1**. The antibodies used for immunohistochemistry.

| **Antibody** | **Dilution Rate** | **Catalog number** | **Manufacturer brand** |
| --- | --- | --- | --- |
| **CSAD** | 1:350 | ab91016 | Abcam |
| **SAT1** | 1:150 | #PA5-106434 | Invitrogen |
| **HDC** | 1:100 | ab37291 | Abcam |
| **CPT1** | 1:800 | ab128568 | Abcam |
| **FAS** | 1:150 | ab99359 | Abcam |

**Supplementary Table S2**. Primer sequences for real-time PCR.

| **Primer** | **Forward** | **Reverse** |
| --- | --- | --- |
| **β-actin** | CGTGCGTGACATCAAAGAGAA | TGGATGCCACAGGATTCCAT |
| **CSAD** | CCAGGACGTGTTTGGGATTGT | CTCCTTCCATTCGCAGACCTT |
| **SAT1** | GAGAACACCCCTTCTACCACT | GCCTCTGTAATCACTCATCACGA |
| **HDC** | CTCGTTGCCTACACCTCTGAT | CTCGGAGTGAGAAGTTGTCGT |
| **CPT1** | ACCACTGGCCGAATGTCAAG | AGCGAGTAGCGCATGGTCAT |
| **FAS** | CCTGGATAGCATTCCGAACCT | AGCACATCTCGAAGGCTACACA |

**Supplementary Table S3**. The potential DKD-associated rate-limiting enzymes in the dysregulated metabolic pathways.

| **Enzyme** | **Full name of the enzyme** | **EC number** | **Pathway** | **Related metabolite(s)** | | **Function** |
| --- | --- | --- | --- | --- | --- | --- |
| **CSAD** | Sulfinoalanine decarboxylase | 4.1.1.29 | Taurine and hypotaurine metabolism | L-Cysteate, Taurine | Catalyzes the biosynthesis of taurine | |
| **SAT1** | Spermidine/spermine N1-acetyl transferase 1 | 2.3.1.57 | Arginine and proline metabolism | Spermidine, Spermine | Catalyzes the acetylation of spermidine and spermine | |
| **HDC** | Histidine decarboxylase | 4.1.1.22 | Histidine metabolism | Histidine, Histamine | Induces the decarboxylation of histidine to form histamine | |
| **CPT1** | Carnitine O-palmitoyl-transferase 1 | 2.3.1.21 | Fatty acid degradation | Palmitoyl-CoA | Facilitates long-chain FA crossing mitochondrial outer membranes | |
| **FAS** | Fatty-acid synthase | 2.3.1.85 | Fatty acid biosynthesis | Long-chain fatty acids | Catalyzes the formation of long-chain FAs | |
